# Successful Management of Pasteurella multocida Pneumonia in a Chronic Obstructive Pulmonary Disease Patient: A Case Report Highlighting the Importance of Tailored Antibiotic Therapy

**DOI:** 10.7759/cureus.60210

**Published:** 2024-05-13

**Authors:** Pratiksha Moliya, Hasan Al-Obaidi, Hussein Harb, Iya A Agha, Farshad Bagheri

**Affiliations:** 1 Internal Medicine, Jamaica Hospital Medical Center, New York, USA; 2 Basic Sciences, Ross University School of Medicine, Bridgetown, BRB; 3 Dermatology, New York Institute of Technology College of Osteopathic Medicine, New York, USA; 4 Infectious Diseases, Jamaica Hospital Medical Center, New York, USA

**Keywords:** pasteurella multocida, infectious disease, chronic obstructive pulmonary disease (copd), copd, individualization of antibiotics treatment, pneumonia

## Abstract

This report describes a patient with *Pasteurella multocida* pneumonia. The patient was a man in his 70s with significant comorbid conditions, including chronic obstructive pulmonary disease (COPD), and is an example of the diverse presentations of *P. multocida* infections increasingly found in the literature. The novelty of this case lies in the manifestation of *P. multocida* pneumonia in a patient with underlying respiratory conditions and its successful management, outlining a unique clinical scenario and a tailored therapeutic approach.

A 71-year-old male with a medical history of COPD, asthma, tremors, hypertension, and arthritis presented to the emergency department with progressive shortness of breath, productive cough, and chest tightness. The initial diagnosis was COPD exacerbation and left lower lobe pneumonia, for which a regimen of ceftriaxone and azithromycin was initiated. The patient's condition was further complicated by the persistence of symptoms. Following sputum culture analysis, *P. multocida* infection was identified. Consequently, the antibiotic regimen was tailored, transitioning the patient to doxycycline, which led to substantial clinical improvement, enabling discharge with a 10-day course of oral doxycycline.

This case elucidates the importance of precise microbiological diagnosis in patients with complex respiratory conditions, as it guides more targeted antibiotic therapy. It highlights the need for clinical vigilance for atypical pathogens like *P. multocida* in patients with COPD exacerbations, especially when conventional treatment strategies yield suboptimal responses. The successful resolution of the pneumonia underscores the effectiveness of antibiotic stewardship guided by sputum culture findings.

## Introduction

*Pasteurella multocida*, a small gram-negative coccobacillus from the *Pasteurellaceae* family lineage, colonizes the upper respiratory and gastrointestinal tracts of an array of wild and domestic mammals [1]. *P. multocida* bears a significant zoonotic characteristic, establishing a consequential health concern for humans. The predominant infections in humans engendered by *Pasteurella* manifest as localized wound infections, typically originating from animal-inflicted bites or scratches. However, the scope of disorders can cover a wide spectrum, causing critical medical conditions, including septic arthritis, meningitis, peritonitis, systemic sepsis, abscess formation, and pneumonia. This amplifies the importance of a thorough understanding of and mitigation strategies concerning the transmission and infection mechanisms of *P. multocida* to limit its potential health detriments and fortify public health infrastructures [1,2]. Pasteurellosis can be attributed to exposure from licks on existing skin abrasions or contact with mucous secretions from household pets. The respiratory tract is the second most prevalent site for *P. multocida* infection, with *P. multocida* predominantly acknowledged as a commensal organism among individuals with chronic pulmonary ailments. Nevertheless, specific instances of pulmonary pasteurellosis can escalate into severe respiratory tract infections, which potentially have fatal consequences [1-3].

## Case presentation

A 71-year-old male with a medical history of chronic obstructive pulmonary disease (COPD), requiring 3 liters per minute of supplemental oxygen, and other comorbidities, including asthma, tremors, hypertension, and arthritis, presented to the emergency department. The patient reported experiencing progressive shortness of breath, a productive cough with white sputum, and chest tightness, persisting for three days. The patient reported having two cats in the household.

Physical examination and initial investigations

Upon physical examination, rales were noted. Initial vitals in the emergency room (ER) were recorded as follows: blood pressure (BP) of 124/60 mmHg, heart rate (HR) of 108 beats per minute, respiratory rate (RR) of 24 breaths per minute, saturation of oxygen of 95% on 3 L of oxygen, and a temperature (T) of 98°F. The patient was placed on bi-level positive airway pressure (BiPAP) due to respiratory distress. Labs were significant for slightly elevated troponin at 0.05. Arterial blood gas analysis showed a pH of 7.43, arterial partial pressure of carbon dioxide (PCO2) of 53.0 (H), arterial partial pressure of oxygen (PO2) of 89.0, arterial bicarbonate (HCO3) of 35.2 (H), and arterial base excess of 8.7 (H). WBC count was 8K, with slightly elevated CRP.

Diagnosis and hospital admission

Based on the clinical presentation, physical examination, and initial investigations, a diagnosis of COPD exacerbation was made. The imaging findings also indicated mild bronchiectasis with bronchial plugging and patchy pleural-based densities, suggesting a possible concurrent infection or another pulmonary pathology. Subsequently, the patient was admitted for further management and treatment of COPD exacerbation.

Therapeutic intervention

**Figure 1:**
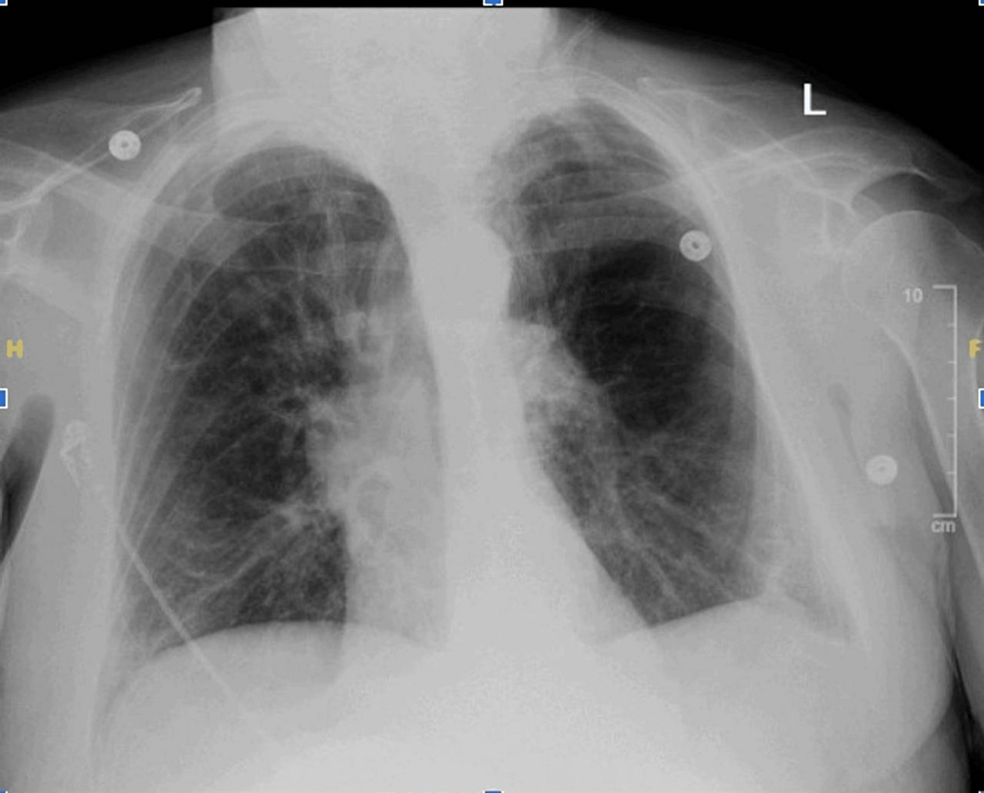
Chest X-ray showing prominent markings present in both lungs, with a small region of atelectasis/infiltrate in the left lower lobe.

Upon admission, the patient was initially started on ceftriaxone and azithromycin. We sent a sputum culture for analysis. A chest X-ray (Figure 1) identified a left lower lobe pneumonia. Thus, the same antibiotic regimen was continued.

During the hospital stay, one dose of vancomycin was administered, and cefepime was added to the regimen due to continued sputum production. The sputum culture results later revealed the presence of *Pasteurella multocida*. Following identifying *P. multocida*, the initial antibiotics were tapered off, and the patient was transitioned to doxycycline.

Follow-up

After the antibiotic adjustment, the patient showed significant improvement. He was discharged on oral doxycycline 100 mg PO (per os, by mouth) to be taken for 10 days.

## Discussion

The organisms belonging to the *Pasteurella* group present themselves as coccoid to small Gram-negative entities, demonstrating a facultative anaerobic nature while also being non-motile and non-spore-forming, with sizes ranging from 1 to 2 μm in length. *P. multocida* exists as a "normal flora" in the oropharyngeal cavity of domestic and wild animals such as cats, dogs, rats, horses, rabbits, and pigs [3-5].

Primarily, *Pasteurella multocida* triggers infections in the skin and soft tissue. Cases of primary lung diseases often result from inhaling airborne contaminants originating from infected nasopharyngeal secretions of pets. Clinical signs linked to *P. multocida* vary from a cough, which may or may not be accompanied by hemoptysis or chest discomfort, to severe debilitation. This is notably evident in older groups with pre-existing lung conditions such as chronic obstructive pulmonary disease and bronchiectasis [6].

A penetrative injury is not a requisite for disease transmission, as instances of *Pasteurella* osteitis and meningitis have been documented following mere licks from carrier animals [7]. The emergence of respiratory and other invasive infections (e.g., bacteremia, meningitis, and endocarditis) is infrequent, predominantly manifesting in immunocompromised individuals, the elderly, neonates, or those afflicted with chronic pulmonary disease [8,9].

Within human hosts, *P. multocida* is culpable for various diseases. *P. multocida* can cause respiratory infections, fowl cholera, wound infections, cellulitis, and septicemia in non-human animals and humans. The isolation and identification of this bacterium are straightforward, attributed to its proficient growth on both blood and chocolate agar mediums [10,11].

Transmission of *Pasteurella* to humans chiefly occurs through direct contact with animal secretions, facilitated by actions such as biting, scratching, licking, or kissing. However, the history of such exposures cannot always be determined. The elderly patient was probably exposed to the secretions from his multiple pets via inhalation of contaminated aerosols, possibly leading to colonization of the tracheobronchial tree and subsequent pneumonia [12].

Reports indicate that prevalent infections caused by *P. multocida* are localized wound infections that arise from animal bites or scratches, typically presenting with a swift onset of pain, erythema, swelling, and fever [6]. The respiratory tract is the second most common infection site [1]. In a cohort study of 108 patients suffering from *Pasteurella* pleuropulmonary infections, 49 were identified with pneumonia, 37 with tracheobronchitis, 25 with empyema, and three with lung abscesses. The occurrence of *P. multocida* infections was relatively low in this study, with 13 cases identified over 12 years, where respiratory tract infections were the most common. *P. multocida* pneumonia cases predominantly occur in older individuals with underlying chronic pulmonary disorders. A review conducted by Hubbert and Rosen on 136 patients with *P. multocida* infections unrelated to animal bites reported that 80 cases involved the respiratory tract, with all patients having chronic pulmonary diseases [13].

The exact incidence of *P. multocida* respiratory tract infections in routine clinical practice remains elusive, given the low likelihood of identifying the causative pathogen in community-acquired pneumonia. The causative pathogen is identified in less than 6% of outpatients and 25% of inpatients, with even lower rates in upper respiratory tract infection cases. Isolation of *P. multocida* from the respiratory tract poses a challenge. Despite the presumed high occurrence, the incidence of bacteremic pneumonia attributable to *Pasteurella* spp. remains unclear, due to the lack of ample large-scale sample groups in studies [14]. However, in a cohort of 49 patients with pneumonia, bacteremia was detected in 55% of the patients [15].

The primary treatment advocated for *P. multocida* infections is administration of penicillin [10,13]. However, the emergence of penicillin-resistant *P. multocida* strains has been documented [16]. In light of such resistance, the use of alternative therapeutic agents, including second and third-generation cephalosporins and tetracyclines, has been recommended [16].

Recently, in 2023, a potent vaccine against *P. multocida *has been developed that utilizes recombinant PMT-NC protein antigen with adjuvants such as recombinant suilysin (Sly) or CpG oligodeoxynucleotides to boost immunogenicity. Testing in piglets significantly reduced symptoms post-*P. multocida* challenge, showing potential for bivalent vaccine development in combating atrophic rhinitis [17].

A mortality rate nearing 30% has been associated with patients with *P. multocida* infections accompanied by bacteremia, pneumonia, meningitis, arthritis, or peritonitis. Such cases typically necessitate an extended course of antibiotic treatment to achieve a favorable clinical outcome [18].

A report by Kofteridis et al. illustrated a *P. multocida* infection manifested as pneumonia in a patient with underlying COPD and other comorbid conditions. The initial broad-spectrum antibiotic coverage was essential and might have prevented further complications. However, the specific identification of *P. multocida* allowed for a targeted antibiotic approach with doxycycline, leading to the patient's improvement and eventual discharge. The switch to a more targeted antibiotic represented prudent antibiotic stewardship and a personalized therapeutic approach that resulted in a favorable outcome for the patient [14].

This case presentation described a *P. multocida*-induced pneumonia in a 41-year-old zoo worker and pet owner. Notably, despite the standard treatment route of penicillin for *P. multocida* infections, this patient was successfully treated with amoxicillin-clavulanate, showcasing an effective alternative treatment option. The prompt alleviation of symptoms post treatment underscores the importance of accurate and timely diagnosis, which in this case was facilitated by thorough lab workup and bronchoalveolar lavage fluid cultures. This case also highlights individuals' potential occupational hazards in close contact with animals. It reiterates the significance of detailed patient history and examination in reaching a correct diagnosis, especially in the absence of overt animal bites or scratches [5].

In our case of an older adult in his 70s with COPD developing *Pasteurella multocida* pneumonia, several critical facets deserve discussion. Our decision for initial therapeutic intervention entailed administering broad-spectrum antibiotics ceftriaxone and azithromycin for presumed bacterial pneumonia. After we identified *P. multocida* in the sputum culture, the antibiotic regimen was changed to doxycycline. The optimal treatment duration for *P. multocida* infections and the timing of medicines still needs to be discovered. Typically, mild soft-tissue conditions necessitate seven to 10 days of oral treatment. More severe instances may warrant a treatment period of 10 to 14 days. Deep-tissue conditions frequently call for four to six weeks of treatment, with intravenous therapy being a possible initial approach [19].

Potential risks of doxycycline use include diarrhea or vomiting, oral or vaginal thrush, rash or itching, changes in nail appearance, mild esophagus irritation, loss of taste, and persistent ear ringing. In our case, none of these complications were observed [20].

The patient's successful transition to oral doxycycline and subsequent discharge illuminates the value of an individualized treatment approach, particularly in multifaceted clinical presentations. A structured follow-up plan is advisable to monitor the patient's respiratory status, check for potential recurrence of *P. multocida* infection, and ensure adherence to COPD management protocols. Since *P. multocida* infection is often tied to animal exposures, counseling on minimizing such exposures and reviewing the patient's living environment might be prudent to avert infection recurrence.

The case reiterates the significance of meticulous microbiological diagnosis, individualized treatment planning, and possibly a multidisciplinary approach involving pulmonology, infectious disease, and other relevant specialties for comprehensive patient management. It contributes valuable insights into managing complex respiratory infections, especially in patients with significant comorbid conditions, thereby broadening the understanding of *P. multocida* infections amidst chronic respiratory diseases.

## Conclusions

Our case demonstrates the importance of a precise microbiological diagnosis, especially in patients harboring complex respiratory conditions, to foster a more targeted antibiotic intervention. The manifestation of *P. multocida* pneumonia in a COPD patient underscores the necessity of clinical vigilance toward atypical pathogens when conventional treatment approaches yield suboptimal responses. The therapeutic transition to doxycycline upon confirmation of *P. multocida* infection played a pivotal role in the patient’s recovery, demonstrating the potential benefits of antibiotic stewardship steered by accurate sputum culture analyses. However, the stipulated 10-day doxycycline regimen was empirically determined; the optimal duration remains unclear. Hence, it beckons a further investigative endeavor to delineate the precise period for doxycycline therapy in managing *P. multocida* pneumonia, which would crucially contribute toward honing the treatment protocols for such intricate clinical scenarios.
